# Developing immortal cell lines from *Xenopus* embryos*,* four novel cell lines derived from *Xenopus tropicalis*

**DOI:** 10.1098/rsob.220089

**Published:** 2022-07-06

**Authors:** Gary J. Gorbsky, John R. Daum, Hem Sapkota, Katja Summala, Hitoshi Yoshida, Constantin Georgescu, Jonathan D. Wren, Leonid Peshkin, Marko E. Horb

**Affiliations:** ^1^ Cell Cycle and Cancer Biology Research Program, Oklahoma Medical Research Foundation, Oklahoma City, OK, USA; ^2^ Genes and Human Disease Research Program, Oklahoma Medical Research Foundation, Oklahoma City, OK, USA; ^3^ Depatment of Cell Biology, University of Oklahoma Health Sciences Center, Oklahoma City, OK, USA; ^4^ National Xenopus Resource and Eugene Bell Center for Regeneration Biology and Tissue Engineering, Marine Biological Laboratory, Woods Hole, MA, USA; ^5^ Department of Systems Biology, Harvard Medical School, Boston, MA, USA

**Keywords:** *Xenopus*, embryo, development, cell line, cell cloning, anura

## Abstract

The diploid anuran *Xenopus tropicalis* has emerged as a key research model in cell and developmental biology. To enhance the usefulness of this species, we developed methods for generating immortal cell lines from Nigerian strain (NXR_1018, RRID:SCR_013731) *X. tropicalis* embryos. We generated 14 cell lines that were propagated for several months. We selected four morphologically distinct lines, XTN-6, XTN-8, XTN-10 and XTN-12 for further characterization. Karyotype analysis revealed that three of the lines, XTN-8, XTN-10 and XTN-12 were primarily diploid. XTN-6 cultures showed a consistent mixed population of diploid cells, cells with chromosome 8 trisomy, and cells containing a tetraploid content of chromosomes. The lines were propagated using conventional culture methods as adherent cultures at 30°C in a simple, diluted L-15 medium containing fetal bovine serum without use of a high CO_2_ incubator. Transcriptome analysis indicated that the four lines were distinct lineages. These methods will be useful in the generation of cell lines from normal and mutant strains of *X. tropicalis* as well as other species of *Xenopus*.

## Graphical abstract

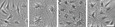

## 1. Introduction

The speed and economy of modern genome, epigenome, transcriptome and proteomic analyses provide unparalleled opportunity to benefit from specific experimental advantages of existing and emerging model organisms. For *Xenopus laevis* and *Xenopus tropicalis*, perhaps the most common amphibian model species, advantages include large clutches of embryos and external development making these organisms powerful systems for study of vertebrate development and physiology. *Xenopus* share with other vertebrates a large percentage of their genes and large regions of synteny with humans [1]. Inbred lines of animals of the two species have been developed and their genomes sequenced and annotated (www.xenbase.org). Of the two species in most common use, *Xenopus laevis* is more widely known. The use of *X. laevis* for genetic manipulations is somewhat complicated by the fact that it is an allotetraploid species, evolutionarily derived from the crossing of two related, ancestral species. As a result, many genes (approx. 56%) are expressed from two sets of alleles [2]. By contrast, *Xenopus tropicalis*, is a true diploid organism. Detailed developmental analyses of gene expression during early *Xenopus* development are available [3,4]. Modern approaches using TALEN and CRISPR technologies have been used successfully to edit genes in embryos of both *X. laevis* and *X. tropicalis* [5–8].

The use of cell lines in mammalian research is often the efficient, front-line approach for mapping gene regulators and biochemical pathways as well as for manipulating genes and studying drug responses. Cell lines have many advantages for exploring biochemical pathways, including simplifying molecular analyses, simple physiological manipulation through application of drugs, and accessibility for high-resolution microscopic imaging. Specifically, the tractability of *Xenopus* cell culture makes *Xenopus* cell lines ideal for research and teaching laboratories with minimal equipment and expense. Typically, *Xenopus* cell cultures are grown in simple incubators or at room temperature using a culture medium (70% L15 medium, 20% H_2_O and 10% serum). L15 medium does not require an enriched CO_2_ atmosphere. *Xenopus* cells will grow robustly in a desk drawer. Like any tissue culture, propagation of *Xenopus* cell lines requires sterile technique, but since they are unlikely to harbour pathogens of danger to humans, they do not require BSL2 precautions. Finally, *Xenopus* cell lines may eventually serve as an efficient conduit for studying developmental biology pathways that are more rapidly productive than similar work with embryos. With cell lines as the basic unit of genetic manipulation, as opposed to the current methods of embryo injection, specific gene manipulations can be selected in cell clones and fully characterized at the molecular level. Mutant cell lines are easily frozen and can be stored indefinitely, at low cost, as frozen stocks. Potentially, somatic cell nuclear transfer will allow the production of mutant frogs in the first (F0) generation using nuclei from the cell lines, negating the need for breeding.

The advantages of cell lines hold true for *Xenopus*, but current cell lines are limited, particularly for the diploid *X. tropicalis.* Over twenty cell lines derived from *X. laevis* adult or tadpole tissues are listed in the Cellosaurus database (https://web.expasy.org/cellosaurus), including X-C, XTC, A6, XL177, XL2, XL58 and XLK-WG. X-C cells and XL177 were reported to be aneuploid [9,10]. XL2 cells were described to contain two cell populations with chromosome numbers of 36 (the euploid number in *X. laevis*) and 74 [11]. Chromosome numbers in other *X. laevis* cell lines have not been reported. We have studied mechanisms of cell division and mitotic cell cycle control in cells in culture using the *X. laevis* S3 cell line [12]. This line was developed in the laboratory of Dr Douglas Desimone at the University of Virginia. Chromosome spreads prepared from S3 cells show normal ploidy (G.J.G. *et al*. 2022, unpublished observations). For *X. tropicalis* (euploid chromosome content of 20), only one permanent cell line has been described. This line, termed Speedy, has a stable aneuploid karyotype of 21 chromosomes being trisomic for chromosome 10 [13]. Here we describe the development and characterization of four novel cell lines, three with normal ploidy, developed from embryos from the inbred Nigerian strain of *X. tropicalis*.

## 2. Methods

### 2.1. Establishment of primary cultures

*Xenopus tropicalis* Nigerian strain (NXR_1018, RRID:SCR_013731) embryos were produced by *in vitro* fertilization then dejellied with cysteine using standard protocols [14]. Embryos were reared in shallow dishes in 0.1× Marc's Modified Ringer's Solution (MMR) at 30°C. Twenty healthy stage 19 embryos were transferred to a 1.5 ml sterile microfuge tube, moved to a horizontal laminar flow hood and rinsed 5 times with 1.5 ml of 0.1× MMR containing gentamycin (final concentration 5 µg ml^−1^ from 10 mg ml^−1^ stock, Gibco), penicillin/streptomycin (1× from 100× stock, Mediatech) and Fungizone (final concentration 0.5 µg ml^−1^ from 250 µg ml^−1^ stock, Gibco). Embryos were then rinsed once with sterile phosphate buffered saline containing the same concentrations of gentamycin, penicillin/streptomycin and fungizone (PBS+). Embryos, still in their vitelline envelopes, were then treated briefly, approximately 10–15 s, with 70% ETOH with 3 to 4 inversions of the tube. The 70% ETOH was quickly removed and replaced with PBS+. Then the embryos were rinsed four times with PBS+.

After the last rinse, the PBS+ was replaced with Trypsin-EDTA (final concentration 0.25× from 10× stock, Atlanta Biologicals). The embryos were disrupted by gentle pipetting, and flicking of the tube. Too vigorous pipetting at this stage resulted in unacceptable levels of cell disruption. The embryos were incubated in the Trypsin-EDTA solution for 4 min with occasional inversion of the tube. Tubes containing the cells were centrifuged at 240×g for two min to pellet most cells but leave yolk platelets released from broken cells in the supernatant. The supernatant was removed, and the cells were gently resuspended in *Xenopus* cell culture medium (70% calcium-free L15 medium (US Biologicals, cat. no. L2101-02 Leibovitz L-15 Medium w/L-Glutamine, Calcium-Free (Powder)), 20% H_2_O, 10% fetal bovine serum with 1× penicillin/streptomycin). Tubes were centrifuged once more at 240×g for 2 min, the supernatant discarded, and the cells resuspended in culture medium, then plated into a 60 mm tissue culture dish with low calcium L15-3G medium.

L15-3G medium was prepared as follows. Conditioned medium was prepared by growing cells from the *Xenopus laevis* S3 cell line in 75 cm^2^ tissue culture flasks. After 2–3 days of culture, medium was collected from these cells and centrifuged at room temperature on a clinical centrifuge at 520×g to pellet debris. Originally, the medium was then collected into a sterile bottle and kept at 4°C. Subsequently, adding the medium to a bottle stored at −20°C was found to preserve growth-promoting activity for longer times. When 200–400 ml of conditioned medium had been collected, it was thawed and sterile filtered through a 0.2 µm filter using vacuum filtration. The sterile medium was distributed to 50 ml sterile tubes (40 ml per tube) and placed at −20°C until use. When needed a tube was thawed, centrifuged at 520×g to pellet any precipitates formed during freezing and thawing. The conditioned medium was mixed 1 : 1 with fresh medium and supplemented with 2 ng ml^−1^ basic fibroblast growth factor and 20 ng ml^−1^ epidermal growth factor. This medium was termed L15-3G and stored at 4°C.

After initial plating of the cells, dishes were sealed with plate sealing tape then incubated overnight at 30°C to allow viable cells to attach and spread. The next day the medium was removed, the dish rinsed gently with PBS to remove unattached cells and yolk platelets released from lysed cells. Fresh L15-3G medium was added to the dish, and the dish was resealed with tape and returned to the incubator. The culture medium was exchanged two times per week.

### 2.2. Growth and cloning of cell lines

Cells were allowed to proliferate until the dish was 80–90% confluent. For passage, cells were resuspended with full strength Trypsin-EDTA. To generate clones, cells were plated at very low density (approx. 100 cells) in 100 mm tissue culture dishes and sealed with tape. Cultures were observed daily to identify clones of proliferating cells derived from single progenitor cells. Clones were marked on the bottom of the dish with a diamond scribe objective and then circled on the bottom of the dish with a marker. Once colonies had grown to size of approximately 500–1000 cells, they were individually treated with Trypsin-EDTA and transferred to a 48-well tissue culture dish. To accomplish this, medium was withdrawn from the 100 mm plate and the plate was rinsed once with PBS. The PBS was removed and sterile stainless steel washers with 4 mm openings were placed over each colony. Trypsin-EDTA was added to the center of each washer and plates were observed until cells had rounded. At that point, most of the Trypsin-EDTA was carefully removed, and the cells were suspended in 30 µl of L15-3G medium. The cells were then transferred to wells of a 48-well dish and fed with L15-3G medium. The outermost wells of the 48-well dish were not used for cells but were filled with sterile water as were any other extra wells not used for cells. Water-filled wells helped to minimize evaporation of medium in wells containing cells. The plates were sealed with plate sealing tape then placed at 30°C. Wells with successfully growing cultures were transferred to 24 well dishes, then 6 well dishes, to 25 cm^2^ flasks and then to 75 cm^2^ flasks. The flasks used were plug sealed and kept securely closed to prevent evaporation. When cell lines were growing vigorously, the L15-3G medium was replaced with *Xenopus* low calcium L15 medium with 10% FBS and pen/strep that did not include conditioned medium or growth factors. Media were exchanged with fresh media every 3–4 days. A protocol for cell line maintenance is provided in the electronic supplementary material.

### 2.3. Karyotype analysis

Cell cultures at 60–70% confluency in 75 cm^2^ flasks were treated with 0.5 uM PD-166285 (inhibitor of Myt1 and Wee1 kinases) and 100 ng ml^−1^ nocodazole (microtubule depolymerizer) for 3 h to enrich the mitotic cell population. Cultures were then treated with Trypsin-EDTA to release the cells from the culture flask, resuspended in 5 ml culture medium and centrifuged at 200×g for 4 min. Cells were resuspended in 1 ml of culture medium. 500 ul of the cell suspension was distributed to two 1.5 ml microfuge tubes and centrifuged at 200×g for 3 min. The medium was aspirated and cells were resuspended in swelling buffer, which consisted of 60% deionized H_2_O, 40% medium. Cells were incubated at 25°C for 15–20 min. Tubes were gently inverted several times to resuspend any cells that had settled. One ml of freshly made 3 : 1 methanol:acetic acid fixative was added dropwise to each tube, which was then kept undisturbed at room temperature for 15 min. Cells were centrifuged at 200×g for 5 min. The fixative was aspirated, and the cells resuspended in 1 ml fixative. Cells were again centrifuged at 200×g for 5 min. All but 30–40 ul of fixative was removed, and the cells were resuspended in the residual fixative by flicking the bottom of the tube. One hundred ul of fixative was then added to each tube and the cells were mixed using a 200 ul pipettor fitted with a wide bore tip.

Ethanol-washed 22 mm coverslips were placed at the bottom of a 15 cm Petri dish on top of wetted filter paper. One to two drops of cell suspension in fixative were dropped onto each coverslip from a height of approximately 45 cm. After each coverslip was used, it was immediately transferred to the bench top on top of H_2_O-wetted kimwipes, and the coverslips were allowed to dry for 1 h or more. Coverslips were transferred to 6 well culture dishes and rehydrated with MilliQ water and labelled in H_2_O for 5 min with DAPI (Sigma-Aldrich, Cat no. 9542) 1 : 10 000 dilution of 1 mg ml^−1^ aqueous stock and Syber Gold 1 : 20 000 dilution of DMSO stock (ThermoFisher, Cat no. S11494). The coverslips were then rinsed with H_2_O 3 times for 4 min each. The coverslips were then mounted on slides with 8 ul of Vectashield and the edges sealed with clear nail polish. Slides were observed and imaged with a Axioplan II microscope using a 100× 1.4 NA objective, a Hamamatsu ORCA ER camera and Metamorph imaging software.

### 2.4. Growth analysis and plating efficiency

Cells were seeded in multiple wells of 6 well dishes and grown in growth medium at 30°C. Cells were seeded on day 0 and grown till day 7. Each day, with the exception of day 5, one well was trypsinized, and the cells were counted by hemacytometer. To test plating efficiency, cells were trypsinized, seeded at initial densities of 500, 1000 and 1500 cells per well in 6 well dishes, and incubated for 10 days. On day ten the cells were rinsed briefly in PBS then stained with 2% Methylene blue in 50% ethanol. They were washed 2× with water and visible colonies were counted.

For analysis of expression of senescence-associated beta galactosidase, XTN-12 cells were plated on 25 mm coverslips in 6 well dishes at approximately 1000 cells per well. The cells were cultured for 7 days then processed for beta-galactosidase expression using a senescence detection kit (BioVision, cat no. K320-250) according to the manufacturer's directions.

### 2.5. RNA sequencing and analysis

For RNA isolation cells were grown in T75 flasks until 70–80% confluent, then trypsinized to create a single cell suspension. RNA was isolated using an RNeasy Mini Kit (Qiagen, cat no. 74134) according to the manufacturer's directions. Prior to RNA-seq analysis quality control measures was implemented as described previously [15]. The concentration of RNA was ascertained via fluorometric analysis on a Thermo Fisher Qubit fluorometer. The overall quality of RNA was verified using an Agilent Tapestation instrument. Following initial quality control steps, sequencing libraries were generated using the Illumina Truseq Stranded mRNA with Library prep kit according to the manufacturer's protocol. Briefly, mature mRNA was enriched for via pull down with beads coated with oligo-dT homopolymers. The mRNA molecules were then chemically fragmented, and the first strand of cDNA was generated using random primers. Following RNAse digestion, the second strand of cDNA was generated replacing dTTP in the reaction mix with dUTP. Double-stranded cDNA then underwent adenylation of 3′ ends following ligation of Illumina-specific adapter sequences. Subsequent PCR enrichment of ligated products further selected for those strands not incorporating dUTP, leading to strand-specific sequencing libraries. Final libraries for each sample were assayed on the Agilent Tapestation for appropriate size and quantity. These libraries were then pooled in equimolar amounts as ascertained via fluorometric analyses. Final pools were absolutely quantified using qPCR on a Roche LightCycler 480 instrument with Kapa Biosystems Illumina Library Quantification reagents.

For RNA-seq, paired-end 75 bp read sequencing was performed at 4 time points on an Illumina NextSeq 500 sequencing platform. Raw reads, in a FASTQ format, were trimmed of residual adaptor sequences using the Scythe software. Low-quality bases at the beginning and end of reads were removed using Sickle, then the quality of remaining sequences was confirmed with FastQC. Further processing of quality sequencing reads was performed with utilities provided by the Tuxedo Suite software. Reads were aligned to the *Xenopus tropicalis* v. 9.0 genome reference using the TopHat component.

### 2.6. Generation cells expressing EGFP-tubulin

Cells were collected by trypsinization and suspension in low calcium L15 medium, (and cell concentrations determined via a Countess FL II automated cell counter (Invitrogen). Between 3.5 and 5.5 × 10^5^ cells per cell line were centrifuged at 200×g for 5 min at room temperature. Supernatants were aspirated and pellets were suspended in 100 ul buffer L solution (Lonza). One microgram of pEGFP-Tub, a plasmid coding for expression using the cytomegalovirus promoter of humanized enhanced green fluorescent protein (EGFP) conjugated to human alpha-tubulin (BD Bioscience/Clontech), was added to each cell suspension. Cell suspensions were transferred to electroporation cuvettes and nucleofection via an Amaxa Nucleofector I (Amaxa) proceeded using the T-30 setting. Cells were transferred to wells of a 6-well tissue culture plate containing 3 ml media. Based on analysis with the fluorescence microscope, we routinely obtained 30–70% transfection efficiencies with 10 to 50% cell survival. (We initially tested lipid transfection reagents but these showed very poor efficiency.) Approximately two weeks post nucleofection, cells from expanded cultures exhibiting GFP fluorescence were collected via fluorescence-activated cell sorting (BD Biosciences, FACSAria IIIu) and cultured.

### 2.7. Microscopy

Karyotype imaging was carried out with a Zeiss Axioplan 2 microscope. Images were captured with an ORCA-ER camera (Hamamatsu) and processed with Metamorph software (Molecular Devices). For Spinning disc confocal images, cells were plated in wells of glass-bottomed Nunc Lab-Tek I Chambered Cover Glass (Thermo Fisher Scientific) in complete low calcium *Xenopus* L-15 media. On the day of imaging the culture media was exchanged to 70% Leibovitz's L-15 media lacking phenol red (Thermo Fisher Scientific) supplemented with 10% FBS, Pen/Strep, mixed at a 7 : 3 ratio with OptiPrep (STEMCELL Technologies) prior to imaging. Fluorescence images acquired at room temperature using a Zeiss AxioObserver inverted microscope equipped with a 63X objective, a confocal Yokogawa CSU-22 spinning disc, and paired with a Hamamatsu ORCA-Flash 4.0 LT and Slidebook software (Intelligent Imaging Innovations). Time lapse images were single plane. Z projection images were collected in 0.1 um Z-steps. Image processing was carried out with Slidebook and Metamorph software.

For light sheet images, cells were plated in wells of an IBIDI glass-bottomed 4 well µ-Slide (IBIDI) in complete low calcium *Xenopus* L-15 media a day prior to imaging and incubated overnight in a humidified chamber at 30°C. Culture media was exchanged to 70% Leibovitz's L-15 media lacking phenol red supplemented with 10% FBS, Pen/Strep, mixed at a 7 : 3 ratio with OptiPrep prior to imaging. Fluorescence images were acquired with a Tilt light sheet (Mizar Imaging) through a Plan-NEOFLAUR N.A. 1.3 100× oil immersion objective (Zeiss) mounted on a Zeiss Axiovert 200 M microscope paired with a Hamamatsu ORCA-ER cooled CCD camera. Metamorph software controlled the microscope system components and image acquisition parameters, and was used for image preparation.

## 3. Results

### 3.1. Pitfalls in establishing stable cell lines from *Xenopus tropicalis* embryos

One of the most significant difficulties in establishing stable cell lines from *Xenopus* embryos is creating and maintaining stringent sterile conditions for the cultured cells. Surface sterilization procedures can be applied during the dejellying process [14]. However, these are generally insufficient to guarantee sterility within cultures of the dissociated embryo cells. Therefore, our protocols emphasize the use of antibiotics during dissociation and, just before dissociation, treatment of embryos within the intact fertilization envelope with 70% ethanol, all within the confines of a sterile culture hood. These precautions are usually, though not always, sufficient to guarantee sterility. Fungal contaminants do occasionally occur but are not common enough to necessitate routine use of fungicides in the culture medium.

A second difficulty encountered during embryo dissociation is disruption of cells resulting in release of numerous yolk platelets that can restrict cell attachment to the culture surface. As noted in the methods, at the stage of embryo dissociation, vigorous pipetting is to be avoided. Contamination of the cultures by released yolk platelets is minimized by differential centrifugation steps. If large numbers of free yolk platelets remain after cells have attached to the substratum, they can be removed the next day by gentle pipetting, discarding the released platelets with the medium and refreshing the culture with fresh medium. Initially many attached cells will contain internal yolk platelets. These will decrease over time in culture and eventually disappear.

Once cells attach, they will begin to proliferate. However, if cells are plated at too low a density, many will undergo a terminal differentiation or senescence. This can be reduced but not eliminated by the use of conditioned medium. Thus, in the initial stages it is beneficial to maintain primary cultures at high density, preferably with 25% to 50% of the substrate occupied by cells. Then when the primary cultures become near confluent, transfer no less than 25% of the resuspended cells to new culture flasks. Eventually robust cell growth will occur and cells can be passaged at greater dilution and even cloned though not all cells plated at very low concentration for cloning purposes will generate successful colonies.

### 3.2. *Xenopus tropicalis* cell lines

**Figure 1.**
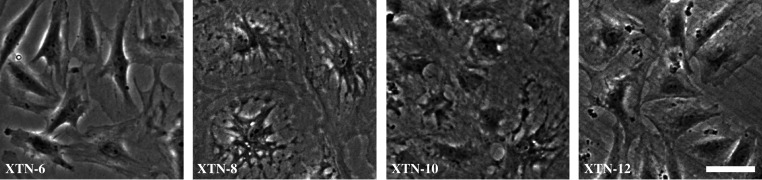
Phase-contrast images of four selected *Xenopus tropicalis* cell lines. Bar = 50 µm.

We established 14 cloned cell lines that were passaged in culture for over six months. Some of the lines appeared morphologically similar suggesting that they were multiple isolates of identical or closely related cells. Based on distinct morphologies, we selected four lines for more detailed analysis (figure 1). We named these cell lines with the prefix XTN, which stands for *Xenopus tropicalis* Nigerian strain*.* The lines selected were named XTN-6, XTN-8, XTN-10 and XTN-12.

### 3.3. Karyotype analysis

**Figure 2.**
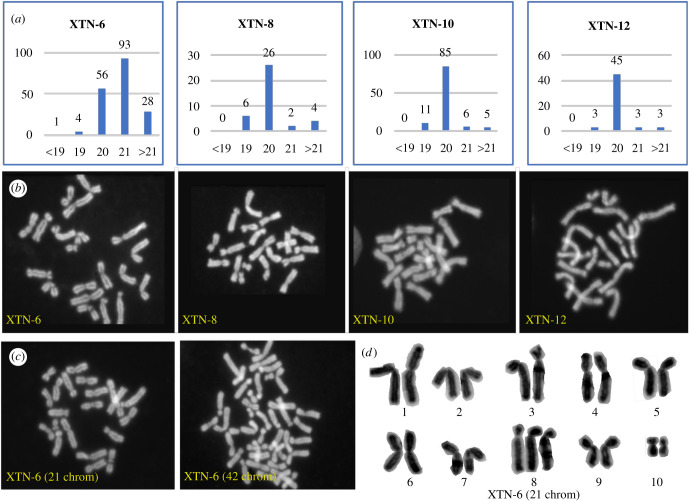
Karyotype analysis of four selected *X. tropicalis* cell lines. (*a*) Quantitation of chromosome spreads from XTN-6, XTN-8, XTN-10 and XTN-12 cell lines. Karyotype categories are listed on the X axis and numbers of spreads of each category are listed above the bars. (*b*) Examples of normal diploid spreads (20 chromosomes) from each cell line. (*c*) Chromosome spreads from XTN-6 cells showing single examples of 21 chromosomes, single chromosome aneuploidy and 42 chromosomes, possibly a tetraploid cell derived from a cell which originally contained 21 chromosomes. (*d*) An example of a karyotype analysis which revealed that XTN-6 cells with 21 chromosomes contain an extra copy of chromosome 8. (Chromosomes are numbered according to the updated nomenclature [16]).

Chromosome spreads prepared from the four selected *X. tropicalis* cell lines revealed predominantly diploid karyotypes of 20 chromosomes for XTN-8, XTN-10 and XTN-12 cell lines while cells from the XTN-6 line revealed more complex features (figure 2). Most XTN-6 cells contained 21 chromosomes although a significant number showed the normal complement of 20. Chromosome spreads of XTN-6 cells also revealed a large percentage (41.3%) of the cells with more than 21 chromosomes were tetraploid containing 40 to 42 chromosomes. Detailed analysis revealed that the extra chromosome in XTN-6 cells with 21 chromosomes was not a random aneuploidy but reflected a conserved trisomy of chromosome 8.

### 3.4. Growth characteristics

**Figure 3.**
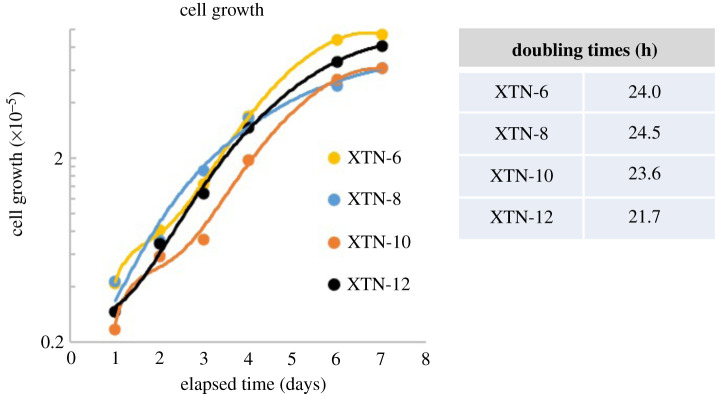
Growth properties of four *X. tropicalis* cell lines.

Cells seeded in a six-well dish at day 0 grew exponentially until day 6 when they began to plateau (figure 3). During exponential growth XTN-6, XTN-8 and XTN-10 cells showed a doubling time of approximately 24 h at 30°C. XTN-12 cells grew slightly faster with a doubling time of about 22 h. We also tested the plating efficiency, namely their ability to form colonies when cells were plated at low density in six well dishes. Generally, cloning efficiency was highest in XTN-8 and XTN-10 cell lines followed by XTN-12 with XTN-6 cells being the least efficient (table 1)

**Table 1.** Plating efficiency of four selected *X. tropicalis* cell lines seeded at low density in 6 well dishes.

no. cells plated	500	1000	1500
XTN-6 colonies formed	6	14	23
XTN-8 colonies formed	98	169	207
XTN-10 colonies formed	24	108	157
XTN-12 colonies formed	28	70	96

**Figure 4.**
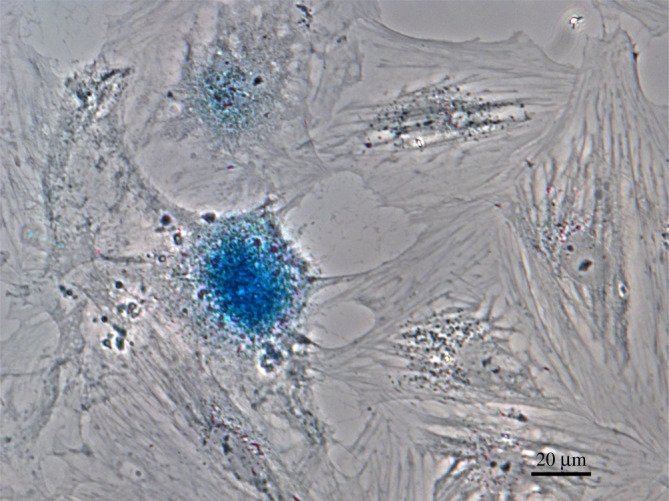
Expression of the senesce marker beta-galactosidase indicated by the blue colour in this phase-contrast image of XTN-12 cells initially plated at low density. Bar = 20 µm.

To examine whether plating efficiency might be hampered by a tendency of cells at low density to undergo senescence, we tested one of the lower efficiency lines, XTN-12, for expression of beta-galactosidase, one of the most common senescence markers [17]. XTN-12 cells plated at low density on coverslips and cultured for 7 days showed cells that were positive for senescence-associated beta-galactosidase, consistent with the interpretation that they had undergone senescence-dependent cell cycle arrest (figure 4).

### 3.5. Transcriptome analysis

**Figure 5.**
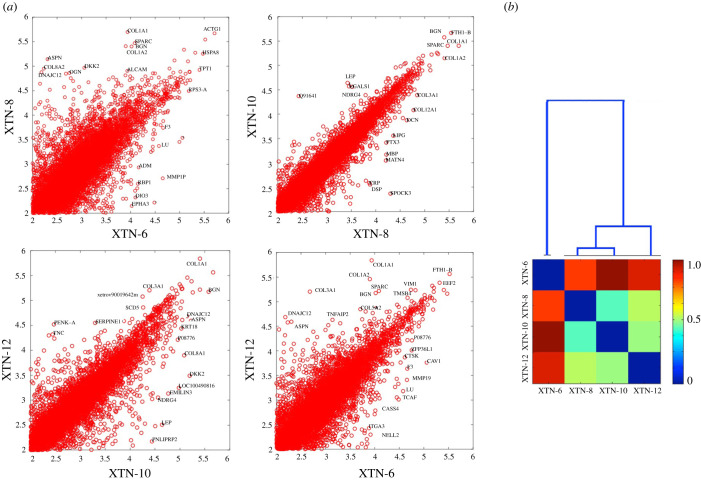
Comparison of gene expression in XTN cell lines. (*a*) Four pair-wise comparisons of gene expression in the cell lines. The most highly expressed genes and outliers are labelled using a human symbol of the homologous gene. It is apparent that XTN-8 and XTN-10 are most similar to each other and XTN-6 is most different from the rest. (*b*) Dendrogram and expression comparing all four cell lines. The numerical scale on the dendrogram reflects similarity matrix using a cosine distance with blue indicating greater similarity and red greater dissimilarity between pairs of lines.

Consistent with morphological differences observed among the four cell lines, gene expression profiles revealed considerable dissimilarities among the lines (figure 5*a*; electronic supplementary material, table S1). As indicated in the dendrogram (figure 5*b*) lines XTN-8 and XTN-10 were most similar to each other and XTN-6 the most distinct. However, a prerequisite for cell line immortality is a mechanism for restoring shortened telomeres generated during DNA replication. All four lines exhibit expression of genes encoding telomerase reverse transcriptase (TERT) and telomerase-associated protein 1 (TEP1), components of the telomerase complex. *X. laevis* embryo expression analyses demonstrate that these genes are also expressed in *X. laevis* embryo stages corresponding to those from which the cell lines were initiated from *X. tropicalis* embryos [4].

To characterize our cell lines further, we compared the gene expression profiles to the single-cell expression data from the *Xenopus tropicalis* embryos profiled around the same developmental stage from which we derived the lines [3]. The bottom-up bulk analysis of the entire transcriptome does not single out any of approximately 200 groups clustered by cell type in that paper (data not shown). We then took a top-down approach focusing on the key marker genes curated from the single-cell data [3] and from *Xenopus* Anatomy Ontology available at Xenbase: https://www.xenbase.org/anatomy/xao.do?method=display&tabId=0). This is presented in electronic supplementary material, table S2 which lists 50 marker genes showing strong differences in expression among the cell lines. Electronic supplementary material, figure S1 shows four pairwise comparisons of expression of the selected markers between two of the lines with the major differentially expressed genes marked. While again we do not see a clear indication of the cell lines expressing a pattern characteristic to just one of the embryonic cell lineages, there are some features that stand out. First, XTN-6 is again the most distinct from the other lines. XTN-6 shows strong differential expression of individual gene markers characteristic of several cell types, endoderm (*gjb2*) and intermediate mesoderm (*osr2*), while the other strong markers for these same cell types are detected in different cell lines—e.g. in XTN-10 for endoderm marker (*c8g*) and XTN-8 for intermediate mesoderm (*tbx1*). XTN-6 shows consistent expression of somite progenitor genes exclusively expressing many markers: *hoxc10*, *hoxc11*, *hoxa11*, *hoxd1*, *hoxd4*, *sp5*.

XTN-8, XTN-10 and XTN-12 show expression of several markers typical for pre-placodal ectoderm (*pitx2, nkx2-3, olfm4, six1, eya2*). XTN-12 expresses collagen 9 isoforms (*col9a1, col9a2 and col9a3*) typical for notochord. All four cell lines show characteristics of the endoderm and the gene program typical for migrating cells (*fli1, snai2, twist1, sox8, sox9*). XTN-6 shows enhanced expression of genes located on chromosome 8, as might be expected given the high proportion of cells trisomic for this chromosome. (Note that this chromosome is annotated as chromosome 10 in Xenbase, consistent with the older chromosome numbering system used for *X. tropicalis* [18].)

### 3.6. Derived cell lines expressing GFP-α-tubulin

**Figure 6.**
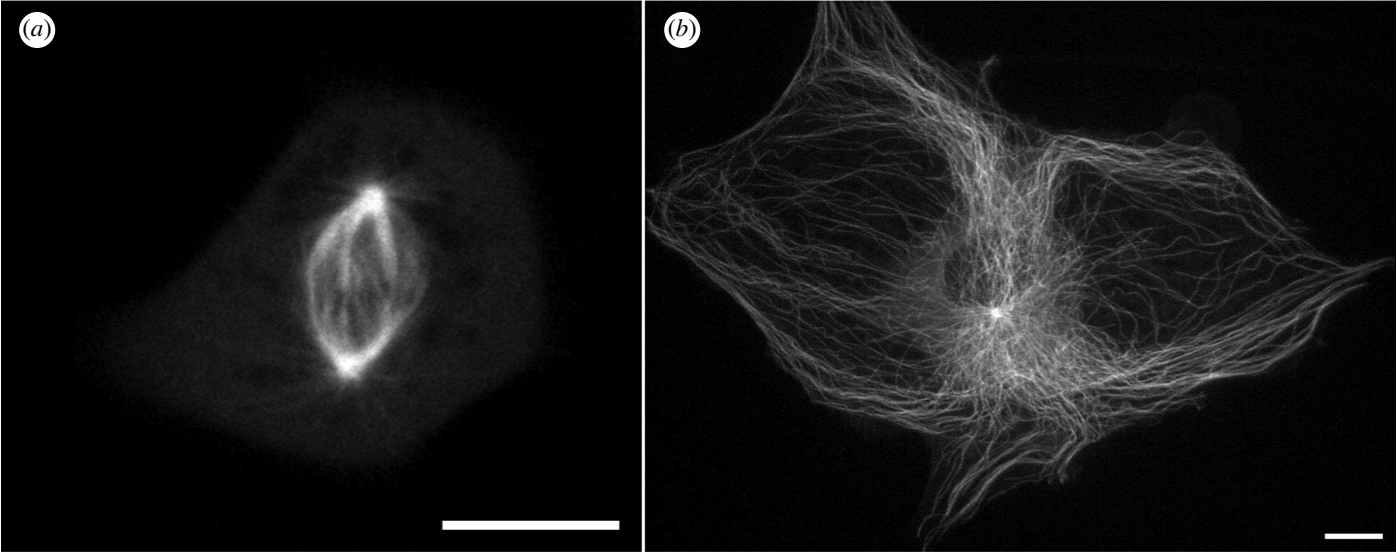
Cells stably expressing EGFP-tubulin imaged with spinning disc confocal microscopy. (*a*) XTN-6 cell in mitosis. Image depicts cell at metaphase captured from a time lapse video recording which is shown in electronic supplementary material, video S1. (*b*) A large XTN-8 cell in interphase. Image depicts a flattened z projection of a live cell. Bars = 10 µm.

**Figure 7.**
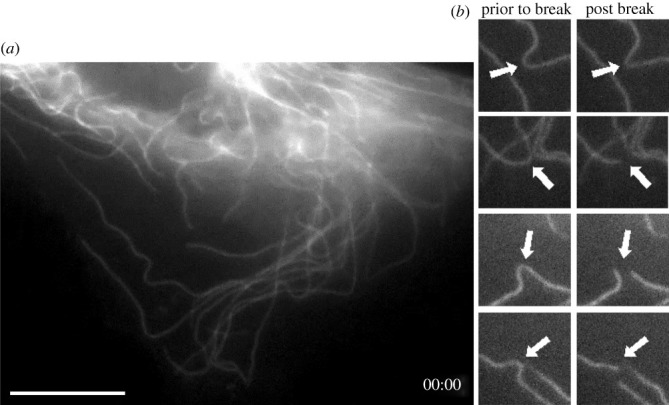
XTN-12 cell expressing EGFP-tubulin imaged with light-sheet microscopy. (*a*) Initial frame from a video centered on the retracting lamella of a moving interphase cell. (*b*) Four instances of microtubule breaks occurring during the video sequence. The entire sequence is available as electronic supplementary material, video S3. Bar = 10 µm.

To explore the usefulness of the newly developed cell lines we transfected cells with transgenes encoding EGFP-α-tubulin. Using antibiotic selection and fluorescence-activated cell sorting, we developed derivative lines stably expressing the transgenes. We then imaged these lines using spinning disc confocal and light-sheet microscopy. Examples of still images of live cells in and individual frames of from video recordings of mitotic and interphase cells are shown in figure 6. Video images of mitotic and interphase microtubule dynamics are shown in electronic supplementary material, videos S1 and S2. Of special note are light sheet images shown in figure 7 and electronic supplementary material, video S3, showing the retracting lamella of an XTN-12 cell with several examples of microtubule breakage and depolymerization. This example of microtubule severing and depolymerization is not due to photodamage from imaging because breakage is not seen in advancing lamellae such as that imaged in electronic supplementary material, video S2. We do not believe that the phenomenon of microtubule severing is restricted to XTN-12 cells since we have occasionally observed severing in other *Xenopus* cell lines. All four lines show strong expression of the genes coding for the microtubule severing complex, katanin and its regulators. Microtubule severing has been previously characterized in mouse fibroblasts and newt lung epithelial cells [19,20].

## 4. Discussion

Elucidating molecular pathways, mapping gene regulators and studying the consequences of mutation and drug treatments is often simplified through use of cell lines in mammalian research. Such advantages hold true for *Xenopus* research as well, but cell line resources have been limited, particularly for the diploid *X. tropicalis.* Here we have developed four new cell lines, three of which are normal ploidy. Morphology and RNAseq differences reveal that the cell lines are likely derived from distinct founder cells from the embryo. Our methods for developing new *Xenopus* cell lines are relatively simple and could be applied to develop lines from the embryos of currently available mutant *Xenopus* animals.

The sexual maturation times of *X. laevis* and *X. tropicalis* are approximately 6–12 months, respectively, making their use in standard genetic crosses problematic. In addition, there is no method for long-term storage of frog eggs, embryos or adults. Frogs must be kept as live animals to maintain a genetic line. Although success has been achieved in freezing *Xenopus* sperm, successful regeneration of a mutant animal line, even under the best circumstances, requires 1–2 years since two generations must be bred. Finally, while transgenesis and gene deletion by TALENs or CRISPR technology is established in *Xenopus*, their current application to fertilized *Xenopus* eggs [3–5] and to *Xenopus* oocytes [2,6,7] generates mosaic embryos and does not allow for selection of specific, defined mutations or gene insertions.

By using *Xenopus* cell lines as the basic unit of genetic manipulation, as opposed to the current methods of embryo injection, specific gene manipulations can be selected in cell clones and fully characterized at the molecular level, prior to generation of the mutant animal. Our cell lines were efficiently transfected with reasonable survival using electroporation with the nucleofector I unit. More recently we have been using a modern instrument, the Thermo-Fisher Neon electroporation device (Thermo-Fisher, cat no. MPK5000) and obtaining near equivalent transfection efficiencies and cell survival, in addition to a simpler protocol. We did not maintain antibiotic selection in the standard long-term culture conditions for our transfected lines. As in many mammalian cells, the proportion of GFP-positive cells in the cultures diminished over time. This necessitated occasionally resorting the cells by FACS to obtain cultures with a high proportion of fluorescent cells.

Although the transfection examples provided above were exogenous transgenes, we anticipate that true gene-editing methods such as those involving TALENS or CRISPR methods should be equally successful in the cell lines since these approaches have been successfully applied with Xenopus embryos [8]. Once generated, mutant cell lines are easily frozen and can be stored indefinitely, at low cost, as frozen stocks. Potentially, somatic cell nuclear transfer methods will allow the production of mutant frogs in the first (F0) generation, negating the need for breeding. Even mutations that might lead to infertility or embryonic lethality can be studied in the F0 generation. However, nuclear transfer efficiency from current cell lines is inefficient (data not shown). This is likely due to difficulty in epigenetic reprogramming of the transferred nuclei. We did not detect substantial expression of stem cell markers in our cell lines. Potentially, using our techniques to produce embryonic stem cells or the development of induced pluripotent stem cells from the current cell lines will greatly increase the efficiency of embryo production after nuclear transplantation.

## Data Availability

RNAseq data are available in electronic supplementary material, table S1 [21].
